# Ketogenic Diet Therapy for the Treatment of Post-encephalitic and Autoimmune-Associated Epilepsies

**DOI:** 10.3389/fneur.2021.624202

**Published:** 2021-06-16

**Authors:** Khalil S. Husari, Mackenzie C. Cervenka

**Affiliations:** Department of Neurology, Johns Hopkins Comprehensive Epilepsy Center, Johns Hopkins University, Baltimore, MD, United States

**Keywords:** modified Atkins diet, encephalitis, autoimmune epilepsy, drug-resistant, status epilepticus, SE

## Abstract

**Introduction:** Acute Encephalitis is associated with a high risk of acute symptomatic seizures, status epilepticus, and remote symptomatic epilepsy. Ketogenic diet therapies (KDT) have been established as a feasible and safe adjunctive management of refractory- and super-refractory status epilepticus. However, the role of KDT in the chronic management of Post-encephalitic epilepsy (PE) and autoimmune-associated epilepsy (AE) is unknown. This study aims to investigate the use of KDT in patients with PE and AE.

**Methods:** A retrospective single-center case series examining adult patients with PE and AE treated with the modified Atkins diet (MAD), a KDT commonly used by adults with drug-resistant epilepsy.

**Results:** Ten patients with PE and AE who were treated with adjunctive MAD were included. Four patients had either confirmed or presumed viral encephalitis, five patients had seronegative AE, and one patient had GAD65 AE. The median latency between starting MAD and onset of encephalitis was 6 years (IQR: 1–10). The median duration of MAD was 10 months (IQR: 3.75–36). Three patients (30%) became seizure-free, one patient (10%) achieved 90% seizure freedom, and three patients (30%) achieved a 50–75% reduction in their baseline seizure frequency, while three patients (30%) had no significant benefit. Overall, seven patients (70%) achieved ≥50% seizure reduction.

**Conclusion:** In addition to its established role in the treatment of RSE, KDT may be a safe and feasible option for the treatment of chronic PE and AE, particularly in those with prior history of SE. Prospective studies are warranted to explore the efficacy of KDT in management of patients with PE and AE.

## Introduction

Acute encephalitis is associated with significant morbidity and mortality, with an estimated annual global incidence of 0.07–12.6 cases per 100,000 (1). It is associated with a high risk of acute symptomatic seizures, status epilepticus, and remote symptomatic epilepsy (2). The risk of post-encephalitic epilepsy (PE) ranges from 10 to 40% across several studies (2–5). Over the past two decades, numerous neural auto-antibodies associated with encephalitis, seizures, and epilepsy have been discovered (6). This has led to a significant increase in the incidence of identified patients with autoimmune encephalitis and epilepsy (7). Autoimmune-associated epilepsy (AE) or autoimmune epilepsy is now recognized as an important etiology in a subset of patients with both acute symptomatic seizures and chronic epilepsy (6, 8–10). In a 2018 study, the incidence and prevalence of autoimmune encephalitis were comparable to those of infectious encephalitis (11). Treatment of patients with AE is comprised of multiple lines of immunotherapy (particularly in the acute phase) and anti-seizure drugs when indicated (6).

Ketogenic diets therapies (KDT) are characterized by a reduced carbohydrate intake along with a relative increase in the proportions of fat and protein to promote fat metabolism (12). Since the early 2000s, KDT variants have been investigated as a potential therapeutic option for adult patients with intractable epilepsy and status epilepticus (12, 13).

There are five types of KDT used in adults: the classic ketogenic diet (CKD), and less restrictive diets including the modified ketogenic diet (MKD), medium chain triglyceride (MCT) oil ketogenic diet, low glycemic index treatment (LGIT), and the modified Atkins diet (MAD) (14). In adult patients, MAD is typically prescribed as a net 20 g/day carbohydrate limit, which is equivalent to a ratio of 1–2:1 of fat to protein and carbohydrates combined (15).

Several reports have demonstrated the efficacy of the classic ketogenic diet in patients with super-refractory status epilepticus (SRSE) of multiple etiologies (16, 17). CKD appears to be particularly beneficial in patients with new-onset refractory status epilepticus (NORSE) and specifically Febrile Infection-Related Epilepsy Syndrome (FIRES) (18–21). While the etiology of NORSE in most patients remains unknown, the most common identifiable etiology is either autoimmune or viral encephalitis (19, 22, 23).

Although several reports have studied the use of KDT in the acute settings of encephalitis and SRSE, no prior studies have evaluated its use in patients with chronic post-encephalitic (PE) and autoimmune-associated (AE) epilepsies. This investigation aims to evaluate the use of MAD in patients with post-infectious and autoimmune-associated epilepsies. We present a case series treated at the Johns Hopkins Adult Epilepsy Diet Center along with a brief review of the literature for comparison.

## Materials and Methods

### Patient Inclusion

This was a retrospective case series performed at the Johns Hopkins Adult Epilepsy Diet Center (AEDC). A prospectively assembled clinical database of all patients evaluated for KDT at the Johns Hopkins Adult Epilepsy Diet Center (AEDC) was approved by the Johns Hopkins Institutional Review Board and all participants or their legally authorized representative provided written consent to be included. Out of this database, we identified adult patients with post-encephalitic (PE) and autoimmune-associated (AE) epilepsies who were evaluated at AEDC from January 2014 to 2020. Patients with childhood (<18 years old) onset of their encephalitis or epilepsy were excluded, those without quantifiable seizures prior to starting MAD, as well as patients who declined participation in the database. PE was defined as persistent seizures after an episode of encephalitis (24), while AE is defined as per the consensus criteria (25). A subset of the patients who met the inclusion and exclusion criteria were previously described in studies evaluating the use of ketogenic diet in super-refractory status epilepticus and new-onset refractory status epilepticus during their acute course while this study focuses on long-term outcomes (16, 17, 19).

### Data Collection and Analysis

The electronic medical records of participants were reviewed to extract demographic information, clinical, radiological, and laboratory data. Response to MAD was assessed based on the reported seizure frequency prior to MAD compared to seizure frequency at the time that the records were reviewed.

### Statistical Analysis

Descriptive analyses of the categorical and continuous data were performed using proportions, frequencies, medians, and ranges. The statistical analyses were performed using Stata16.0 (College Station, TX).

## Results

Fifteen patients with adult-onset PE or AE were evaluated to initiate MAD at the AEDC. Five patients were excluded from analysis for the following reasons: one patient received education on MAD initiation and never started, one patient was lost to follow up, two patients were non-adherent with the diet and stopped it early (within 2–4 weeks), and one patient who was seizure-free on ASDs and was interested in using MAD as adjunctive therapy to potentially lower ASD burden.

Ten patients with PE and AE met the inclusion criteria. Four patients had viral or presumed viral encephalitis (three unknown pathogens and one with California encephalitis), five patients had seronegative AE, and one patient had GAD65 antibody-associated epilepsy. Demographic and clinical characteristics are outlined in Tables 1, 2. The majority of patients presented with an episode of refractory status epilepticus (RSE) (seven patients, 70%). In only three patients (30%), the KDT was started during the acute settings to treat RSE (16, 17, 19).

**Table 1:** Baseline characteristics of patients with post-encephalitic and auto-immune associated epilepsy treated with the modified Atkins diet.

Male, *n* (%)	6 (55%)
**Type of encephalitis**	
Presumed and confirmed viral encephalitis	4 (40%)
Seronegative AE	5 (50%)
GAD65 associated-epilepsy	1 (10%)
Age at encephalitis onset, median (IQR) years	37 (30–42)
Age at epilepsy onset, median (IQR) years	38 (35–42)
Number of ASD used prior to MAD, median (IQR) years	7.5 (6–10)
Age at MAD onset, median (IQR) years	42 (35–50)
Duration between onset of epilepsy and MAD, median (IQR) y	4 (1–8)
Duration between onset of encephalitis and MAD, median (IQR) y	6 (1–10)
Presence of RSE	7 (70%)
KDT started during RSE	3 (30%)
**MRI findings**	
Mesial temporal sclerosis	1 (10%)
T2 FLAIR hyperintensity	5 (50%)
Normal	4 (40%)
**Epilepsy type (out of eight patients)**	
Unifocal temporal	1 (12.5%)
Unifocal Extra-temporal	3 (37.5%)
Multifocal	4 (50%)
**Seizure type**	
Focal aware seizures	3 (30%)
Focal impaired awareness seizures	10 (100%)
Focal to bilateral tonic clonic seizure	4 (40%)

*ASD, anti-seizure drugs; GAD, gamma aminobutyric acid; MAD, modified Atkins diet; IQR, interquartile range; RSE, refractory status epilepticus; KDT, ketogenic diet therapy*.

**Table 2:** Full details of all 10 patients with post-encephalitic and auto-immune associated epilepsy treated with the modified Atkins diet.

**Patient**	**Sex**	**Etiology**	**Age range at encephalitis onset**	**Age range at epilepsy onset**	**Age range at MAD onset**	**RSE**	**KDT started during SE**	**No of ASD prior to MAD**	**No of concurrent ASD**	**Seizures type**	**Epilepsy type**	**MAD duration (months)**	**% of seizure reduction**	**Adverse effects**	**Reason for stopping MAD**
1	F	Viral	25–30	35–40	35–40	N	N	2	1	FIAS, BTC	n/a	24	100	Transient hyperlipidemia	Still on MAD
2	F	Viral	25–30	30–35	30–35	N	N	1	1	FIAS	n/a	3	0	None	Lost follow up
3	M	SN AE	20–25	20–25	25–30	Y	N	8	2	FAS,FIAS, BTC	Left temporal	8	90	None	Restrictive, compliance
4	F	SN AE	45–50	45–50	45–50	Y	N	6	5	FIAS	Bi-temporal	4	0	None	Lack of response
5	M	SN AE	35–40	35–40	45–50	Y	Y	6	4	FIAS	Left FC	12	100	hyperlipidemia	hyperlipidemia
6	M	SN AE	40–45	40–45	40–45	Y	Y	10	4	FIAS	Bi-temporal	79	50	None	Still on MAD
7	F	SN AE	35–40	35–40	35–40	Y	Y	10	4	FIAS	MF, left temporal + Right posterior	6	75	None	Restrictive, compliance
8	M	Viral	35–40	35–40	45–50	Y	N	7	3	FAS, FIAS	Bi-temporal	28	100	None	Still on MAD
9	M	California encephalitis	35–40	35–40	40–45	Y	N	12	3	FIAS, BTC	Posterior extra-temporal	60	75	None	Lost follow up
10	M	GAD associated AE epilepsy	45–50	45–50	55–60	N	N	8	3	FAS, BTC	Extra-temporal (midline seizure)	3	0	None	Lack of response

*SN AE, seronegative Auto-immune encephalitis; GAD, gamma aminobutyric acid; FAS, focal aware seizure; FIAS, focal impaired awareness seizures; BTC, bilateral tonic clonic seizure; MAD, modified Atkins diet; N, No; Y, Yes; n/a, not available*.

The median duration of MAD was 10 months (IQR: 3.75–36). The median age at onset of encephalitis was 37 years (IQR: 30–42). MAD was started at a median of 6 years (IQR: 1–10) after the onset of encephalitis. All 10 patients had focal seizures with impaired awareness, while four patients had additional bilateral tonic-clonic seizures, and three patients had additional focal aware seizures. Eight out of 10 patients had seizures that were localized on EEG. Half of them (four patients) had multifocal epilepsy, three unifocal extra-temporal, and one patient with left temporal localization.

### Seizure Outcomes

Out of the 10 patients, three patients (30%) became seizure-free, and one additional patient (10%) achieved 90% seizure freedom with only rare focal seizures with impaired awareness. Four patients (30%) achieved a 50–75% reduction in their baseline seizure frequency on MAD, while two patients (30%) achieved no benefit. None of the patients had an increased frequency of seizures. Overall, seven patients (70%) achieved ≥50% seizure reduction. Additional benefits of MAD included a decrease in seizure severity (in one patient) and improved concentration and attention (in one patient). In reviewing reported adverse effects, the MAD was generally well-tolerated. One patient had transient hyperlipidemia, while another patient had significant LDL elevation prompting discontinuation of the modified Atkins diet. Three patients remain on MAD at last follow up, while two patients were lost to follow up. Reasons of discontinuation included compliance (two patients) and lack of efficacy (two patients).

## Discussion

Post-encephalitic and autoimmune-associated epilepsies are increasingly recognized etiologies of drug-resistant focal epilepsy (2–5, 8, 10). In this small case series, the modified Atkins diet was safely used by patients with PE and AE and epilepsy with a 70% responder rate (≥50% seizure reduction).

One third of patients in this study achieved seizure freedom, with another 40% achieving 50–90% seizure reduction. MAD was well-tolerated with only one patient having a persistent and treatment-limiting hyperlipidemia. The median on-diet duration was 10 months (IQR: 4–28), suggesting that MAD was feasible to maintain long-term in this patient population. As previously shown in other studies (26), patients who experience improvement in their seizures had longer on-diet duration. Data extrapolated from children suggests that most responders experience a reduction in seizures on KDT within 14 days from initiation of the diet (27). A consensus recommendation from the International Ketogenic Diet Study Group is to continue the KDT for at least a mean of 3.2 months (±1.3 months SD) before assessing its efficacy (14).

The majority of patients in this study (70%) presented with an episode of refractory status epilepticus as an initial presenting symptom. A classic ketogenic diet was first introduced in 30% of patients studied in the setting of super-refractory status epilepticus. Refractory status epilepticus (RSE) occurs when status epilepticus (SE) persists despite the administration of at least 2 parenteral anti-seizure drugs (including an appropriately-dosed benzodiazepine). About 15–22.6% of cases of SE progress to RSE (28, 29). Of those, another 22% continue to progress to SRSE (30) which is defined as SE persisting or recurred after appropriate anesthetic treatment (31, 32). Both RSE and SRSE carry high rates of morbidity and mortality (33). The prevalence of SE in patients with acute encephalitis ranges between 4 and 20% (34–36), while the prevalence of RSE ranges between 24 and 57% (34, 36) and the prevalence of SRSE has not been well-established.

Several prospective and retrospective studies evaluated the use of KDT in the treatment of SRSE of various etiologies (16, 17, 37). In several case series, a classic ketogenic diet (CKD) was effective in abolishing SE in 73–90% of patients (16, 17, 37). A summary of all adult patients with SRSE due to presumed encephalitic and immune-mediated etiologies treated with adjunctive CKD is presented in Table 3. Thirty-four adult patients received CKD, the median age of patients at the onset of SRSE and CKD initiation was 28.5 (21–40). The majority of the patients responded with cessation of SE, suggesting possible benefit, although there were no control groups used for comparison.

**Table 3:** Summary of published adult patients with RSE due to presumed infectious and immune-mediated etiologies treated with adjunctive KDT.

**References**	**KDT type**	**Patients, *n***	**Etiology (*n*)**	**Age (years, range when multiple)**	**Female (%)**	**Time to KDT start (range, days)**	**Time to ketosis (Days)**	**SE resolution, number (%)**	**Time to response (days, range when multiple)**	**Adverse effects**
Wusthoff et al. (38)	CKD	2	RE (1)	24–29	50	20–101	8–10	2 (100)	8–11	None
			VE (1)							
Nam et al. (39)	CKD	1	VE (1)	40	100	15	n/a	1 (100)	30^*^	None
Matsuzono et al. (40)	n/a	1	NORSE (1)	22	0	155	n/a	1 (100)	25–47	None
Thakur et al. (17)	CKD	7	NORSE (4)	23–51 (median = 33)	57	2–60 (median = 24)	1–6	6 (86)	1–31 (median = 2.5)	Acidosis, hyperlipidemia
			NMDA (2)							
			LGI-1 (1)							
Amer et al. (41)	CKD	1	NMDA (1)	21	100	21	n/a	1 (100)	14	n/a
Uchida et al. (42)	n/a	1	NMDA (1)	20	100	n/a	n/a	0^**^	60	None
Cervenka et al. (16)	CKD	6	NORSE (5)	20–55 (median = 38.5)	67	2–39 (median = 14.5)	0–16 (median = 2)	4 (67)	0–8 (median = 3.5)	Metabolic acidosis, hypoglycemia, hyperlipidemia, hyponatremia
			Encephalitis (1)							
Francis et al. (37)	CKD	1	NMDA (1)	21	100	3	2	1 (100) ^¥^	n/a	Acidosis, hypoglycemia, infection, abdomen perforation
Park et al. (20)	CKD	2	FIRES (2)	21–40	50	12–37	n/a	2 (100) ^¥¥^	7	Nausea, vomiting
Prasoppokakorn et al. (43)	CKD, then MCT-KD	1	NORSE (1)	19	100	42	n/a	1 (100)	20	Hyperlipidemia
Noviawaty et al. (44)	CKD	1	NORSE (1)	38	0	49	5	1 (100)^¶^	7	None
Gugger et al. (19)	CKD	10	NORSE (10)	n/a	n/a	12.5 (5–33)^¶¶^	3.5 (1–8)^¶¶^	7 (70%)	14 (4–25)^¶¶^	n/a

* *50% seizure reduction at day 7*,

** *SE resolved after treatment with both stiripentol and KD for 2 months*,

¥ *the patient had protracted course with multiple ASDs added with KD*,

¥¥ *1 patient with >50% seizure reduction*,

¶ *treated with rufinamide, CKD, and VNS*,

¶¶ *median (IQR)*.

*RE, Rasmussen encephalitis; VE, viral encephalitis; NORSE, new-onset refractory status epilepticus; LGI-1, leucine-rich glioma inactivated 1; NMDA, n-methyl-d-aspartate receptor encephalitis; CKD, classic ketogenic diet; VNS, vagal nerve stimulators; n/a, not available; FIRES, Febrile Infection Related Epilepsy Syndrome*.

The management of chronic PE and AE remains challenging with a significant percentage of patients who develop drug-resistant epilepsy and status epilepticus (3, 45). Patients remain refractory despite treatment with immunotherapy and anti-seizure drugs. A retrospective study showed higher seizure freedom rates with sodium channel agents (carbamazepine, phenytoin, oxcarbazepine, and lacosamide) in patients with AE (46), while there was no difference in the seizure relapse rates between valproate and levetiracetam in another retrospective study (3). Patients with PE and AE are more likely to have multi-focal epilepsy and are therefore not ideal epilepsy surgery candidates, and have been shown to have worse epilepsy surgery outcomes when compared to other etiologies of drug-resistant epilepsy (47, 48). More recently, neuromodulation therapy was reported in patients with AE. Feyissa et al. (49) reported four patients with GAD-65 associated epilepsy treated with responsive neurostimulation (RNS) system. Three patients achieved ≥50% seizure reduction and one patient became seizure-free after RNS data-guided temporal lobectomy (49). In addition to immunotherapy, anti-seizure drugs, and neuromodulation, our case series adds ketogenic diet therapy to the list of potential treatments for epilepsy in patients with PE and AE.

Mechanisms by which KDT may be effective management of seizures in AE and PE are under investigation. The exact pathophysiology of chronic AE and PE which leads to seizures has not been fully elucidated (45). It is hypothesized that varying degrees of persistent inflammation, post-encephalitic structural processes (for example mesial temporal sclerosis), or both processes may contribute (45). There is a growing body of evidence supporting the anti-inflammatory properties of KDT (50). Beta-hydroxybutyrate inhibits NLRP3 inflammasome assembly which in turn decreases caspase-1 activation and the release of pro-inflammatory cytokines (51). KDT increases the levels of polyunsaturated fatty acids that can bind and activate the peroxisome proliferator-activated receptors (PPARs) (50). Additionally, the KDT increases adenosine levels in the brain, which has anti-inflammatory effects (52). Moreover, KDT have antioxidant effects (53) and may modulate mitochondrial function by reducing the production of reactive oxygen species (ROS) (54). Therefore, KDT may be effective for the management of seizures in AE and PE not only because of anti-seizure properties but also by anti-inflammatory mechanisms of action, which could explain the high response rate seen in the patients reported here.

Our study have several limitations aside from its retrospective nature. As a quaternary referral center, patients were often treated following their initial presentation and therefore the exact details surrounding the acute encephalitis episode in some patients were not available. Also, two patients were not monitored on EEG to confirm the diagnosis and localization of focal epilepsy which was determined based on clinical presentation. Moreover, the small sample size and the lack of control group might limit the generalizability of our findings. Finally, patients were on anti-seizure drugs, immunotherapy, and other treatments that were not rigorously documented at the time of treatment and may have impacted seizure control independent of KDT.

## Conclusions

In addition to its potential role in the treatment of acute refractory status epilepticus in patients with PE and AE, ketogenic diet therapies may be feasible and safe in the management of chronic post-encephalitic and autoimmune-associated epilepsy. Patients with prior history of SE might respond better to KDT. Further studies are needed to explore the efficacy of KDT in managing seizures in patients with PE and AE. Moreover, whether the early use of KDT can alter the pathophysiology, prognosis, and outcome of patients with encephalitis warrants further exploration.

## Data Availability Statement

The original contributions presented in the study are included in the article/supplementary material, further inquiries can be directed to the corresponding author/s.

## Ethics Statement

The studies involving human participants were reviewed and approved by Johns Hopkins Hospital Institutional Review Board. The patients/participants provided their written informed consent to participate in this study.

## Author Contributions

KH and MC: study design and critical revisions of the manuscript. KH: data collection, analysis, and drafting of the manuscript. Both authors contributed to the article and approved the submitted version.

## Conflict of Interest

MC receives grants from Nutricia, Vitaflo, BrightFocus Foundation, and Army Research Laboratory. Honoraria from American Epilepsy Society, The Neurology Center, Epigenix, LivaNova, and Nutricia. Royalties from Demos/Springer Publishing Company. Consulting for Nutricia, Sage Therapeutics, Glut1 Deficiency Foundation. The remaining author declares that the research was conducted in the absence of any commercial or financial relationships that could be construed as a potential conflict of interest.
